# Supplementary material for: Solubility-Aware Protein Binding Peptide Design Using AlphaFold
Source: Biomedicines. 2022 Jul 7;10(7):1626. doi: 10.3390/biomedicines10071626 (PMC9312799; doi:10.3390/biomedicines10071626)
Supplement: Supplementary file 1 [file biomedicines-10-01626-s001.zip › SupplementaryMaterials1.pdf]

Supplementary Materials

Solubility-aware protein binding peptide design using AlphaFold

Takatsugu Kosugi and Masahito Ohue*

Department of Computer Science, School of Computing, Tokyo Institute of Technology *
Correspondence: ohue@c.titech.ac.jp; Tel.: +81-45-924-5522

1. SUPPLEMENTARY FIGURES

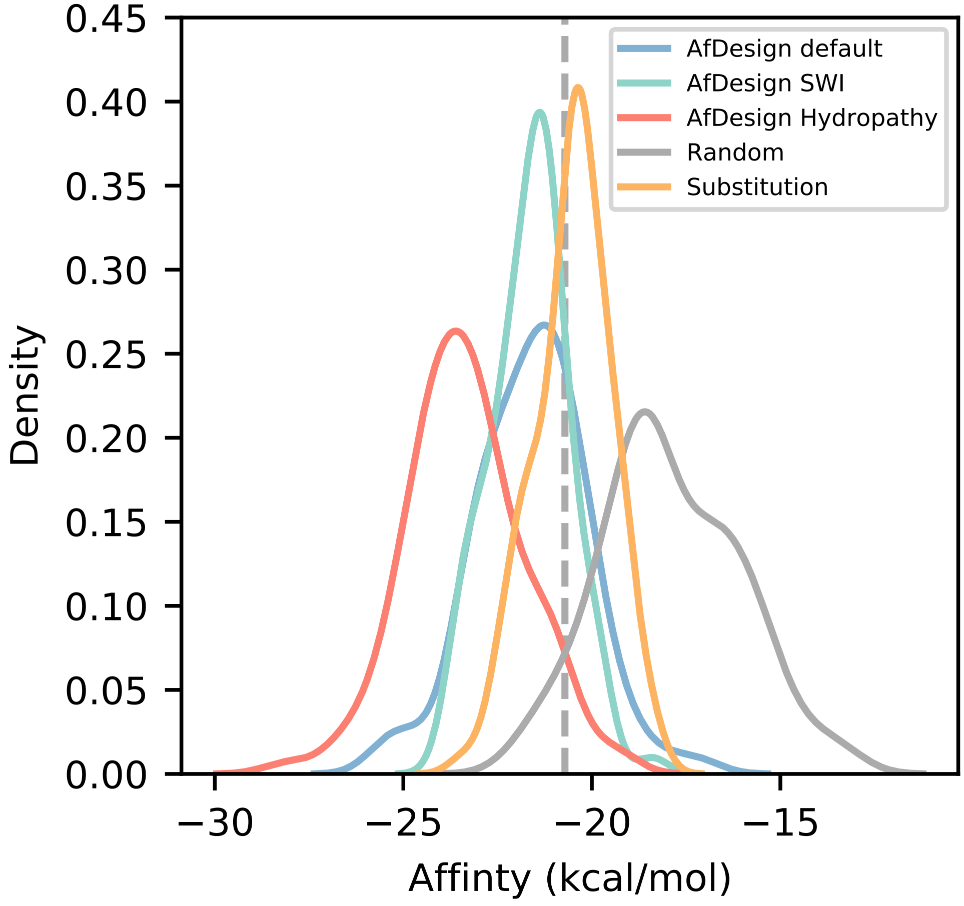

Fig. S1. Comparison of MDM2 binding affinities between random sequences and one residue substitution sequences of p53 peptides and sequences designed by AfDesign. The blue line shows the distribution of binding affinity of peptide sequences designed by default AfDesign, the green line shows the distribution of binding affinity of peptide sequences designed using the Solubility-Weighted Index as a solubility index, and the red line shows the distribution of binding affinity of peptide sequences designed using the Hydropathy Index as a solubility index. The orange line shows the distribution of binding affinity of peptide sequences, the orange line shows the distribution of binding affinity of one residue substitution sequences of p53 peptide sequences, the solid gray line shows the distribution of binding affinity of random sequences, and the dashed gray line shows the binding affinity value of the p53 peptide sequence. All solubility index weights are 0.5.

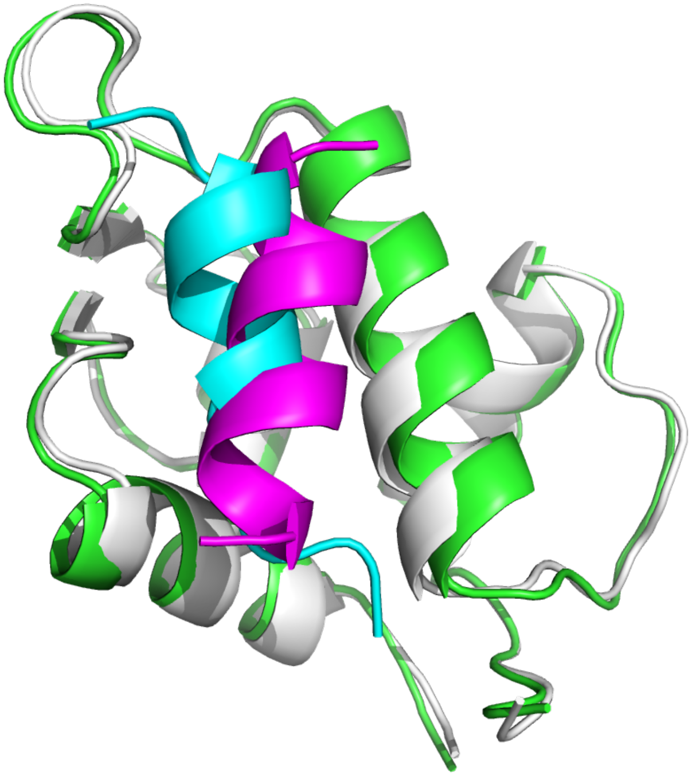

Fig. S2. Comparison of crystal structures and three-dimensional (3D) structures predicted by AfDesign. Green shows the crystal structure of MDM2, cyan shows the crystal structure of the p53 peptide, magenta shows the 3D structure of the sequence designed by AfDesign using the Solubility-Weighted Index as a solubility index with a weight of 0.5 (the sequence with the highest binding affinity and higher logS than that of the p53 peptide), and white shows the MDM2 structure as predicted by AfDesign.

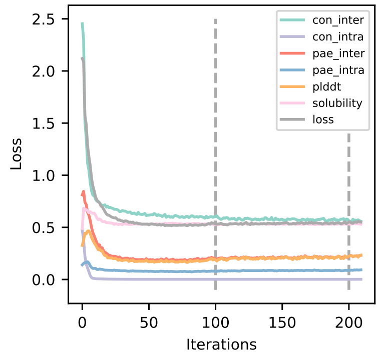

(**a**) Without solubility index

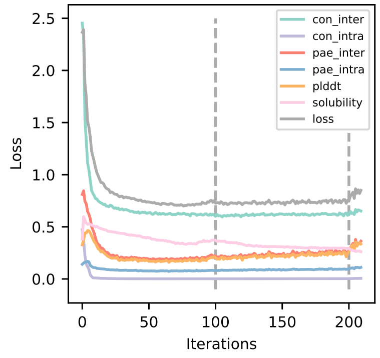

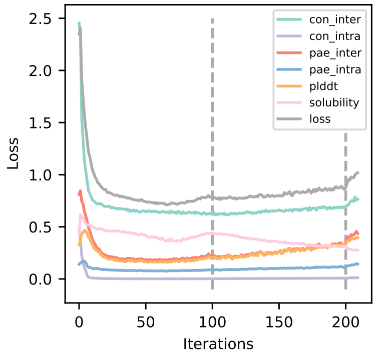

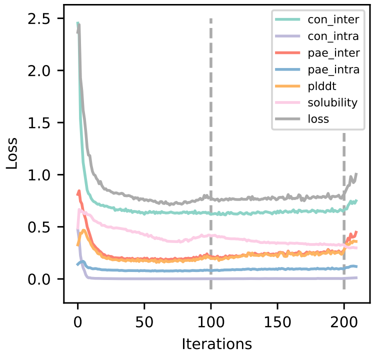

(**b**) Hydrophobicity Index (**c**) Hydropathy Index (**d**) SWI

Fig. S3. Comparison of each solubility index at the median of the AfDesign loss values. The green line shows the median loss value for each iteration of con_inter, the purple line shows the median loss value for each iteration of con_intra, the red line shows the median loss value for each iteration of pae_inter, the blue line shows the median loss value for each iteration of pae_intra, the orange line shows the median loss value for each iteration of plddt, the pink line shows the median loss value for each iteration of solubility, the solid gray line shows the median total loss weighted by the weighted average for each loss, and the dashed gray line shows the stage switching timing (101st, 201st iteration) in design_3stage() of the AfDesing binder hallucination protocol.

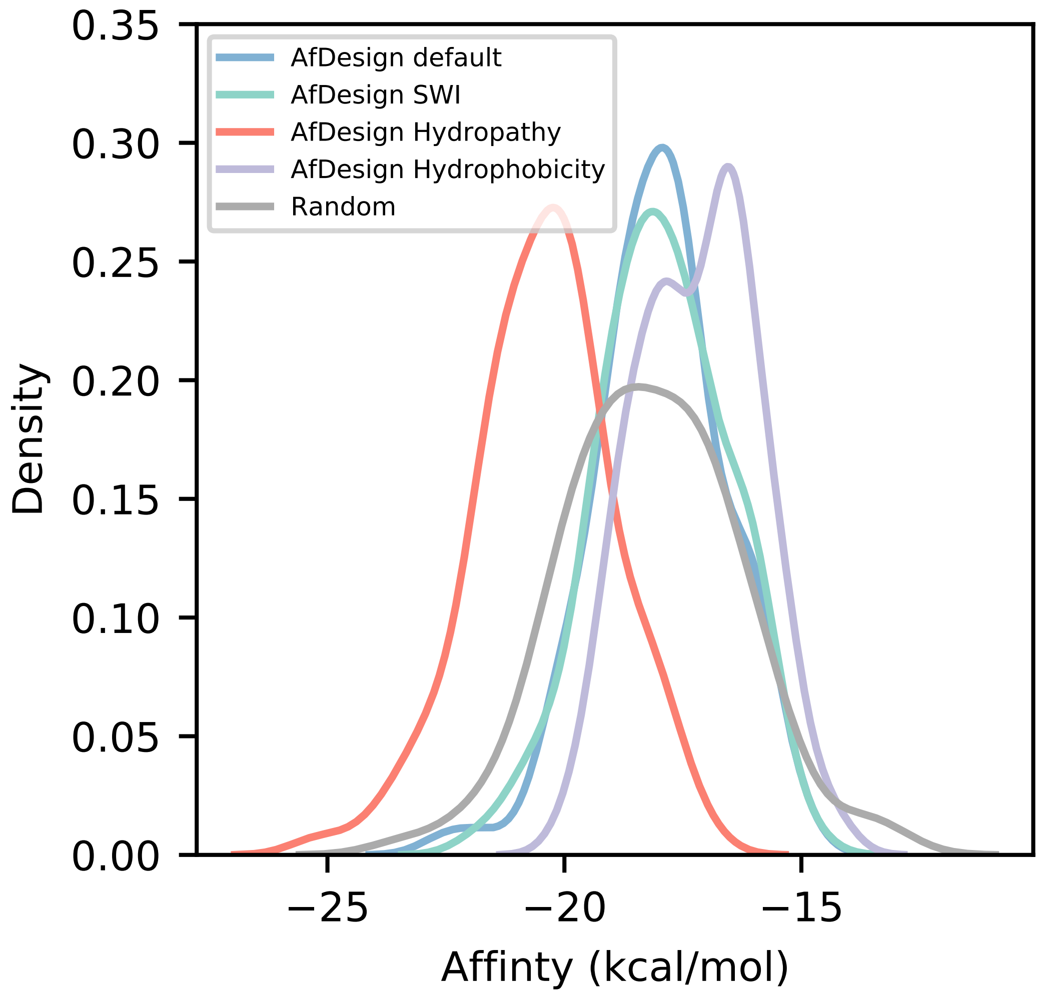

Fig. S4. Comparison of PD-1 binding affinity between random sequences and sequences designed in AfDesign with each solubility index. The blue line shows the distribution of binding affinity of peptide sequences designed by default AfDesign, the green line shows the distribution of binding affinity of peptide sequences designed using the Solubility-Weighted Index as a solubility index, the red line shows the distribution of binding affinity of peptide sequences designed using the Hydropathy Index as a solubility index, the purple line shows the distribution of binding affinity of peptide sequences designed using the Hydrophobicity Index as a solubility index, and the gray line shows the distribution of binding affinity of random sequences. All solubility index weights are 0.5.

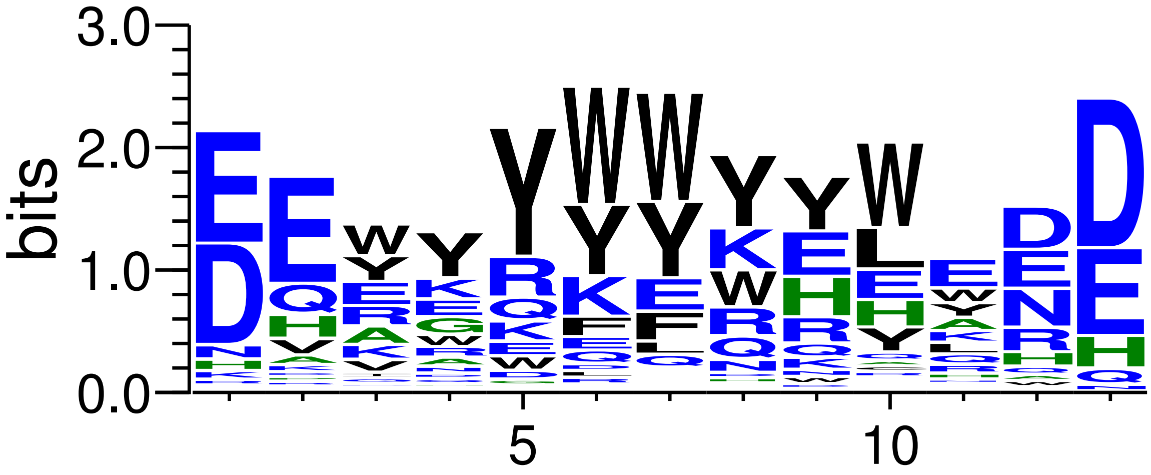

Fig. S5. Weblogo of sequences designed with AfDesign binder hallucination using the Hydropathy Index as a solubility index. Using 90 sequences filtered by logS and binding affinity thresholds for PDIQ + 'N' peptide.

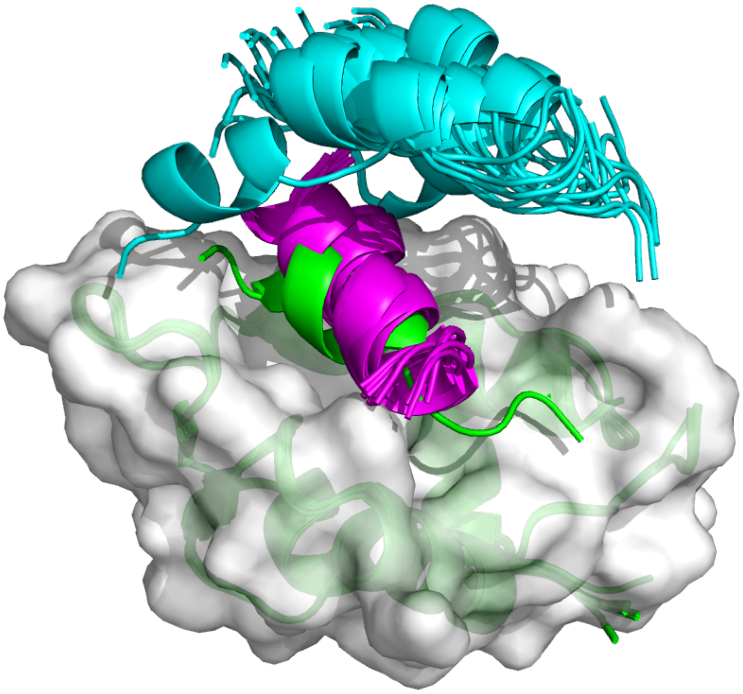

Fig. S6. All top rank models from competitive peptide binding prediction of MDM2 with p53 and our highest affinity peptide consistently indicate the highest affinity peptide as the strong binder. Green indicates native MDM2 and p53 as well as MDM2 in the competitive peptide binding prediction. Cyan indicates p53 in the competitive peptide binding prediction. Magenta indicates the competitor peptide in the competitive peptide binding predictions. The competitor sequence DEVYYWYYHLEND which had the highest binding affinity among the with the filtered 90 sequences.

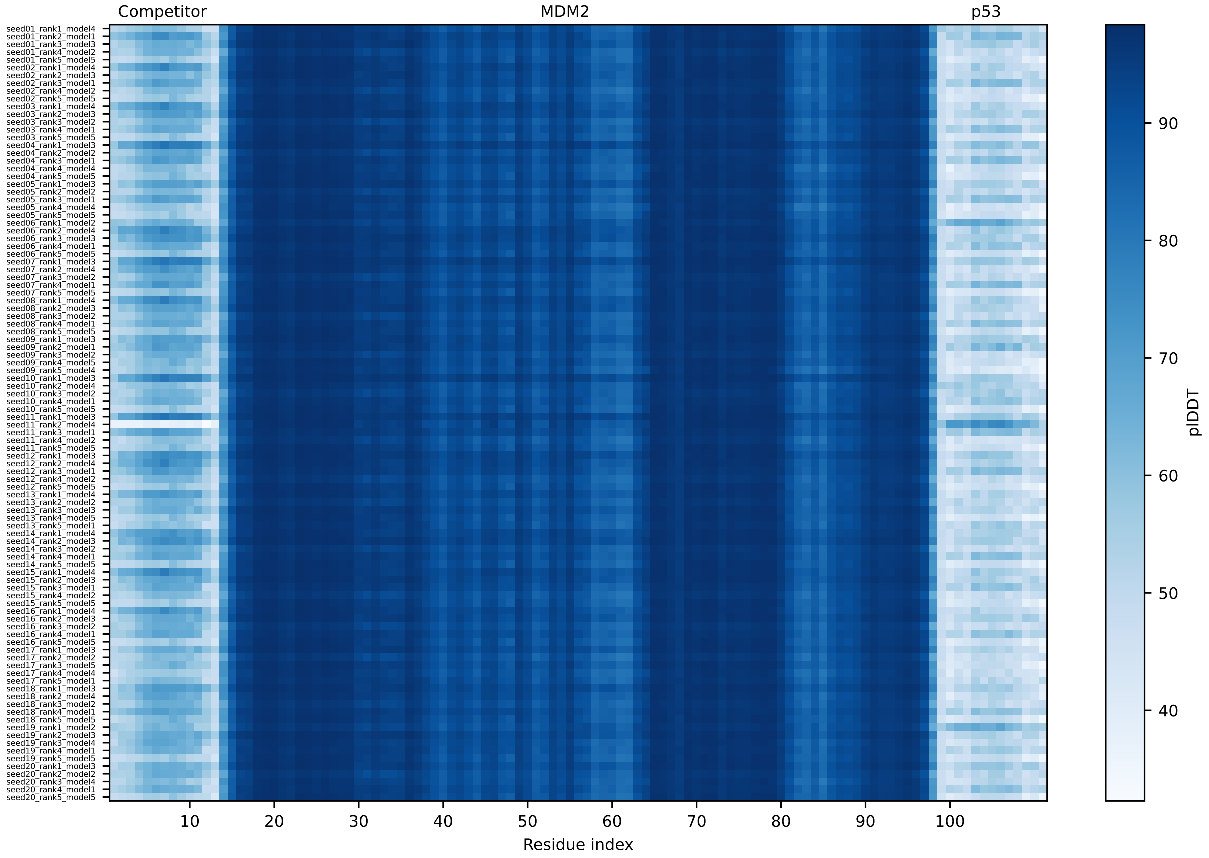

Fig. S7. Heatmap of all pLDDT values in predictions of competitive peptide binding of the competitor peptide and p53 peptide with MDM2 using ColabFold. Our highest affinity competitor peptide was found at the MDM2 binding interface in all cases, except for one prediction. The competitor sequence DEVYYWYYHLEND which had the highest binding affinity among the with the filtered 90 sequences.

2. SUPPLEMENTARY TABLES

Table S1. Values of each solubility index. Values for the Hydrophobicity Index, Hydropathy Index, and Solubility-Weighted Index are referenced from each reference table. The leftmost column shows the one-letter code of amino acids.

	Hydrophobicity Index	Hydropathy Index	Solubility-Weighted Index
A	0.61	1.8	0.835647
R	0.60	-4.5	0.771247
N	0.06	-3.5	0.859743
D	0.46	-3.5	0.907904
C	1.07	2.5	0.520809
Q	0.00	-3.5	0.789435
E	0.47	-3.5	0.987699
G	0.07	-0.4	0.799717
H	0.61	-3.2	0.894791
I	2.22	4.5	0.678412
L	1.53	3.8	0.655422
K	1.15	-3.9	0.926710
M	1.18	1.9	0.629662
F	2.02	2.8	0.584979
P	1.95	-1.6	0.823533
S	0.05	-0.8	0.744091
T	0.05	-0.7	0.809692
W	2.65	-0.9	0.637468
Y	1.88	-1.3	0.611280
V	1.32	4.2	0.735784

Table S2. Normalized solubility indices used as solubility loss for AfDesign. The three solubility indices were normalized to have a maximum of 1 and a minimum of 0, respectively. The Solubility-Weighted Index values were inverted (minus 1) after normalization. The leftmost column show the one-letter code of amino acids.

	Hydrophobicity Index	Hydropathy Index	Solubility-Weighted Index
A	0.230188679	0.700000000	0.325669858
R	0.226415094	0.000000000	0.463603847
N	0.022641509	0.111111111	0.274060271
D	0.173584906	0.111111111	0.170907494
C	0.403773585	0.777777778	1.000000000
Q	0.000000000	0.111111111	0.424648204
E	0.177358491	0.111111111	0.000000000
G	0.026415094	0.455555556	0.402625886
H	0.230188679	0.144444444	0.198993339
I	0.837735849	1.000000000	0.662440832
L	0.577358491	0.922222222	0.711681552
K	0.433962264	0.066666667	0.130628199
M	0.445283019	0.711111111	0.766855148
F	0.762264151	0.811111111	0.862558633
P	0.735849057	0.322222222	0.351616012
S	0.018867925	0.411111111	0.521767440
T	0.018867925	0.422222222	0.381261111
W	1.000000000	0.400000000	0.750136006
Y	0.709433962	0.355555556	0.806226306
V	0.498113208	0.966666667	0.539559639
